# Participation of civil society organizations and academia in COVID-19 governance: insights from a six country study

**DOI:** 10.1093/heapol/czag063

**Published:** 2026-05-16

**Authors:** Sumegha Asthana, Roujia Lin, Sanjana Mukherjee, Alexandra L Phelan, Ibrahim B Gobir, J J Woo, Clare Wenham, Mohammad Mushtuq Husain, Tahmina Shirin, Nevashan Govender, Mohannad Al Nsour, Winifred Ukponu, Adachioma C Ihueze, Sumit Asthana, Renee Vongai Mutare, Claire J Standley

**Affiliations:** Center for Global Health Science and Security, Georgetown University, 3900 Reservoir Road NW, Washington, DC, 20057, United States; PANPREP, PHFI-Institute of Public Health Sciences, Outer Ring Rajendra Nagar, Hyderabad, Telangana, 500030, India; Center for Global Health Science and Security, Georgetown University, 3900 Reservoir Road NW, Washington, DC, 20057, United States; O’Neill Institute for National and Global Health Law, Georgetown University, 600 New Jersey Avenue NW, Washington, DC, 2001, United States; Center for Global Health Policy and Politics, Georgetown University, 3700 Reservoir Road, NW, Washington, DC, 20057, United States; Department of Environmental Health and Engineering, Johns Hopkins Bloomberg School of Public Health, Johns Hopkins University, 615 N Wolfe St, Baltimore, MD, 21205, United States; Johns Hopkins Center for Health Security, Johns Hopkins Bloomberg School of Public Health, Johns Hopkins University, 700 E Pratt St Suite 900, Baltimore, MD, 21202, United States; Georgetown Global Health LTD/GTE Nigeria, No. 2 Babatope Ajakaiye Crescent, Jahi District, Gwarinpa, Abuja, 900108, Nigeria; Center for Global Health Practice and Impact, Georgetown University Medical Centre, 500 First Street NW, Suite 234, Washington, DC, 20007, United States; Lee Kuan Yew School of Public Policy, National University of Singapore, 469C Bukit Timah Road, Singapore, 259772, Singapore; Department of Health Policy, London School of Economics and Political Science, Houghton Street, London, WC2A 2AE, United Kingdom; Institute of Epidemiology, Disease Control and Research (IEDCR), 44 Mohakhali, Dhaka 1212, Bangladesh; Institute of Epidemiology, Disease Control and Research, 44 Mohakhali, Dhaka, 1212, Bangladesh; National Institute for Communicable Diseases, 1 Modderfontien Road, Sandringham, Johannesburg, 2131, South Africa; Eastern Mediterranean Public Health Network, Abdallah Ben Abbas St, Building No. 42, Amman, 11196, Jordan; Georgetown Global Health LTD/GTE Nigeria, No. 2 Babatope Ajakaiye Crescent, Jahi District, Gwarinpa, Abuja, 900108, Nigeria; Independent Consultant, Ontario, Canada; Independent Consultant, Washington, DC, 20007, United States; Center for Global Health Science and Security, Georgetown University, 3900 Reservoir Road NW, Washington, DC, 20057, United States; Center for Global Health Science and Security, Georgetown University, 3900 Reservoir Road NW, Washington, DC, 20057, United States; Heidelberg Institute of Global Health, University of Heidelberg, Im Neuenheimer Feld 672, Heidelberg, 69120, Germany

**Keywords:** decision-making, inclusive governance, multi-sectoral governance, civil society, academia, COVID-19, health emergencies

## Abstract

This paper focuses on the engagement of persons and groups with diverse interests in the governance of the COVID-19 pandemic. During emergencies, involving diverse perspectives in governance mechanisms can contribute to more equitable and evidence-based policies that address societal needs. Previous literature shows that, even with the intent to include diverse perspectives, there can be gaps in engaging non-governmental entities in epidemic governance. This study sought to analyze participation of two important interest groups: academia and civil society organizations (CSOs) during the COVID-19 response, to identify best practices and strategies for enhancing their roles in future health crisis governance. We adopted a comparative case study design to collect data from six countries: Nigeria, Singapore, South Africa, Bangladesh, Jordan, and the United Kingdom, representing varied political and economic contexts, geographic locations, and response strategies during the pandemic. Data sources included key informant interviews and focus group discussions with a total of 64 stakeholders, including government officials, academic experts, and personnel from CSOs. During the COVID-19 pandemic, CSOs actively participated in response efforts, but in the six countries we analyzed, they largely did so independently of the government, particularly during the initial response phase. In some countries, governments involved CSOs only after reports of clusters of new transmission, primarily for risk communication and service delivery like vaccine distribution, without involving them in governance mechanisms like making pandemic preparedness and response policies. Other governments worked with public universities to gather evidence for decision-making and policy formulation starting from the pre-pandemic phase to the initial phase of the pandemic. Across disciplines, experts in epidemiology, mathematical modelling, and infectious diseases were more likely to be consulted, whereas economists, sociologists, ethicists, and anthropologists were less involved.

Key messages

- Current literature indicates gaps in involving non-governmental interest groups, like civil society organizations (CSOs) and academic experts, in pandemic governance.
- CSOs actively participated in pandemic response efforts; however, they primarily worked independently of the government, especially in the initial phase of the pandemic. Gradually, the governments involved CSOs in risk communication and distribution of medical countermeasures, but without sufficient formal mechanisms for them to contribute to governance and decision-making processes.
- Some governments invited public universities to gather evidence for decision-making and policy formulation, starting from the pre-pandemic phase to the initial phase of the pandemic. Across disciplines, experts in epidemiology, mathematical modelling, and infectious diseases were more likely to be consulted.

## Introduction

Governance is defined as the process of making decisions and how those decisions are implemented (or not) (United Nations Economic and Social Commission for Asia and the Pacific, n.d.). Governance encompasses various individuals, institutions, and aspects to tackle public issues and deliver services effectively to the general population (Hyden 2001). It involves joint decision-making of multiple actors and institutions (Chhotray and Stoker 2009). Governance in the health sector refers to ‘a wide range of steering and rule-making related functions carried out by governments and decisions-makers, as they seek to achieve national health policy objectives…’ (WHO, n.d.). It is co-produced by a wide range of actors at the level of the State, such as ministries, parliaments, agencies, authorities and commissions; society, such as businesses, citizens, community groups, global media; and supra-national organisations such as the European Union and the United Nations (Kickbusch and Gleicher 2013).

Health governance during crises differs from routine health governance as it is marked by uncertainty, urgency, fear, and politicization of the crisis (Asthana et al. 2025). Governance of public health emergencies often involves decision-making in short timelines and in the context of incomplete or evolving information (Mukherjee et al. 2023). The COVID-19 pandemic highlighted the need for transformative, agile, inclusive, multi-level, and ethical governance to manage the global crises (Assefa et al. 2022). Participation of diverse interest groups in health crisis governance facilitates knowledge sharing and negotiation of decisions, showcases solidarity around diverse decisions, and ensures transparency of decisions (Gao and Yu 2020, Bhargava 2022). A recent study of COVID-19 taskforces in 24 countries found they included different groups like public, private, academic, and civil organizations. However, the level of involvement of different interest groups varied, and civil society’s participation was notably low. (Rajan et al. 2020). Exclusion of interest groups like civil society organizations (CSOs) and academic experts in health crisis governance risks undermining comprehensive, context-specific, culturally relevant, and effective decision-making (Hossain et al. 2023, Nero et al. 2023, Rajan et al. 2020).

In this analysis, we define governance as health governance co-produced by a diverse group of actors, aligning with the broader discourse on health governance and health crisis governance discussed above. We discuss the participation of two such groups of actors, i.e. CSOs and academics, during COVID-19 as a case of health crisis governance.

CSOs are defined as non-profit, voluntary groups organized at the local, national, or international level, including community groups and non-governmental organizations (NGOs) (United Nations n.d.). Academia includes academic institutions for higher education, which may be non-profit or for-profit, and research organizations providing advisory support and evidence for policy-making, as well as individual experts affiliated with academic/research institutions (Akella 2022). During the COVID-19 pandemic, while politicians took the lead in policymaking, CSOs and academia contributed in diverse ways. Individual scientists with specialist expertise in disease management were invited by some governments to support in the design of policies to combat the virus (Loner et al. 2023). Academia also played an active role in pandemic preparedness and response activities by supplying laboratory services for COVID-19 testing, building capabilities for conducting COVID-19-related research, providing advisory support, and offering evidence for decision-making (Turner et al. 2021). Similarly, CSOs contributed by conducting health awareness activities and providing essential services, including health care and food supply, infection prevention and risk reduction services for the community and frontline staff, social support for vulnerable households to help continue with daily living, and training health care providers in pandemic-related skills (Samat et al. 2022; Abiddin and Ro’is 2023).

The current understanding of the roles of CSOs and academia in COVID-19 management is largely based on expert opinions, with insufficient empirical research to support these insights. Furthermore, although research on pandemic governance highlights the role of civil society to some degree, the involvement of academia in public health decision-making has not been adequately studied. Additionally, our understanding of the role of academia and CSOs in pandemic governance mechanisms across health-systems models and political and economic contexts remains unexplored. Addressing this knowledge gap is essential for future epidemic preparedness and response, as actively involving these interest groups in the governance process is vital for ensuring equitable, evidence-based policies as well as accountability and transparency (Simon 2021).

This paper aims to address the need for a deeper understanding of the roles played by CSOs and academia in managing the COVID-19 pandemic, with an emphasis on their specific roles in governance and decision-making processes related to the pandemic at the national level across six countries. While studying the operational roles played by CSOs and academia in the response was not the central aim of the paper, we sought to explore aspects of this topic, alongside the roles of CSOs/academia in governance structures, to draw insights to enhance future epidemic preparedness and response efforts.

## Methods

### Study design

This study uses a case study approach (Yin 2009) and a comparative research design (Karp et al. 2007) to study the roles of CSOs and academia in the COVID-19 response as well as participation in COVID-19 pandemic governance. Our research design exhibits features close to the comparative research design of the ‘most different systems design’ (Przeworski and Teune 1982), which selects cases that are very different from one another in terms of their structure and context. When a similar pattern appears across these very different cases, it increases confidence in the robustness and reliability of the pattern (Przeworski and Teune 1982). A case study is an empirical inquiry that examines a phenomenon in its real-life context, providing a detailed overview of the phenomenon (Yin 2009). Comparative research design helped us to compare the experience of the governance processes in different settings and allowed us to understand the behaviours of diverse interest groups in governance mechanisms (Karp et al. 2007). This is an exploratory paper that finds a pattern in very diverse countries. We did not intend to produce a rigorous comparative political analysis, but to contribute to the national pandemic governance debate. We purposively selected six countries: Nigeria, Singapore, South Africa, Jordan, Bangladesh, and the United Kingdom (UK) for this study based on their diverse geographical locations, country-income classifications, governance structures, and political regimes.

Nigeria, a low-middle-income country, is a federal republic that adopted a US-style constitution in 1999 after military rule since 1960 (LeVan 2014). Singapore, a high-income country, is a parliamentary republic that has been dominated by the People’s Action Party and functioning as a de facto one-party state since 1959 (Wong and Huang 2011). South Africa, an upper-middle-income country, has been a constitutional democracy since the end of apartheid in 1994 and has adopted a parliamentary executive system (Mattes 2002). Jordan, a lower-middle-income country, is a hereditary monarchy with considerable royal influence. Since 2014, constitutional changes have shifted it from a constitutional to an almost absolute monarchy, with the king playing a significant role in political affairs. (Salameh 2024). Bangladesh, a lower-middle-income country that gained independence in 1971, became a parliamentary republic and shifted to the Westminster system in 1991 after military rule. Despite progress, Bangladesh’s confrontational bipolar political system, marked by electoral violence and challenges in ensuring credible elections, means democratic principles are still not fully implemented (Mozahidul Islam 2015). The UK, a high-income country, is famously the most parliamentary of democracies, with the Westminster parliament lying at the heart of UK democracy (Russell 2021). The Monarchy is a ceremonial symbol uniting the UK, whose sovereignty rests with Parliament and the electorate (Blackburn 2022).

CSOs in Nigeria actively engage in various roles to promote transparency and accountability as well as in developing international codes with governments, corporations, and scholars (Ngara 2019). Academics in Nigeria have historically driven social and political change in Nigeria, challenging authoritarianism and supporting democracy (Asiyanbi and Towoju 2025). During the Ebola outbreak, NGOs helped in organizing and coordinating immediate large-scale response strategies as soon as the outbreak was declared (Ibrahim and Ismail 2016). Civil society in Singapore is largely state-sanctioned, facing a widespread perception that the government is wary of civil society and tends to stifle dissent (Human Rights Watch 2021). Selected academic institutions in Singapore work closely with the government, with various academics having secured positions in the Ministry of Health (MOH) to advise the government from time to time (Asthana et al. 2024). Scholars note that the Singapore government did not partner effectively with NGOs during the 2003 SARS outbreak (Kim et al. 2022). CSOs in South Africa have supported governments in the previous HIV/AIDS epidemic, particularly in providing care to people living with HIV/AIDS during the epidemic (Coutinho et al. 2012). However, their influence in South Africa’s democratic framework has reportedly diminished, and civil society has been reduced to fulfil the requirements and interests of the state, donors and big business, rather than addressing the concerns of citizens (Leonard 2014). The Jordanian government provided limited opportunities for CSOs to grow, and often existing CSOs were embedded in bureaucracy and legal codes so the government could regulate and monitor them (Wiktorowicz 2000). However, Jordanian CSOs display significant agency by reframing donor discourse, building coalitions, and engaging with justice concerns (Hussein 2026). CSOs, NGOs, and community organizations in Bangladesh have played a key role in advocating for human rights, social justice, and government accountability (Afroze et al. 2025). Political liberalization in the 1990s enabled greater involvement in governance and development; however, CSOs are not consistently able to play the role of both independent monitor and advocate of the government (Schurmann and Mahmud 2009). For past epidemics such as avian influenza, the government has partnered with NGOs and other non-governmental partners (Rimi et al. 2019). The government–civil society relationship in the UK is fragile, with CSOs seen as manageable rather than equal partners. A recent report calls for a genuine two-way partnership to harness civil society’s insights and innovation for government goals (Hamida et al. 2025).

Given this substantial variation in governance structures, political trajectories, institutional capacities, and socio-economic contexts across the selected countries, a direct, attribute-by-attribute comparison of decision-making processes was not methodologically feasible. Therefore, the study uses a broader analytical framework to compare governance mechanisms and participation levels of key actors, allowing for a systematic cross-case analysis that respects contextual and structural differences without forcing artificial equivalence.

### Data collection

#### Sources of data and methods of data collection

A combination of desk reviews of documents, remote and in-person key informant interviews (KIIs), and focus group discussions (FGDs) were used to collect data for the study. Through the desk review, we identified relevant policy statements, guidelines, government reports, and journal articles. This data provided country context, and identified potential key informants for KIIs and FGDs. Participants were purposively sampled to represent a wide range of organizational affiliations, experiences, and views of the COVID-19 pandemic response in the six selected countries. In total, 64 individuals agreed to participate in a KII or FGD. A full list of participant types, primary data collection method, and data collection timeline for each country is provided in Table 1. Verbal informed consent was obtained from all participants at the start of the interview or FGD. KIIs and FGDs were conducted in English and recorded for transcription purposes. Each interview took ∼30–60 min, while the FGDs took ∼120 min. To maintain participant anonymity, all responses were de-identified unless participants provided permission for their names and affiliations to be used. To maintain participant confidentiality, all respondents were kept anonymous and given a unique identifier using the country code and numerical value.

**Table 1:** List of types and geographic distribution of participants included for key informant interviews and focus group discussions.

Country	Data collection timeline	Method of primary data collection	Type of participant	Number of participants
Nigeria	June 2022–September 2022	Key informant interviews	Federal government officials	7
			State government officials	5
			Civil society organization representatives	5
			Academic experts	3
			Development partner representatives	1
		Focus group discussion	Civil society organization representatives	4
Singapore	June 2022–September 2022	Key informant interviews	Government officials	8
			Civil society organization representatives	1
			Academic experts	3
		Focus group discussion	Academic experts	3
South Africa	January 2023–March 2023	Key informant interviews	Government officials	4
			Academic experts	3
UK	September 2022−June 2023	Key informant interviews	Government officials	2
			Academic experts	4
Bangladesh	June 2023−September 2023	Key informant interviews	Government officials	2
			Academic experts	3
Jordan	July 2023−September 2023	Focus group discussion	Civil society organization representatives	4
		Key informant interviews	Academic experts	2
Total				64

#### Data analysis

KIIs and FGDs were transcribed using Otter.ai software (Mountain View, CA, USA) and checked for reliability and completeness. Three independent researchers, Sumegha A., R.L., and Sumit A., then analysed the transcripts, coding data into themes, sub-themes, and codes related to the extent and characterization of participation. The study’s primary analytical method drew inspiration from grounded theory and employed an iterative process comprising multiple rounds of coding. The process began with descriptive or open coding, followed by focused and axial coding (Vollstedt and Rezat 2019). The first round of open coding involved breaking down the data as codes describing the process of governance, such as creation of task forces, allocation of resources and responsibilities, and consultations with subject-matter experts and other non-state interest holders. The second round of axial coding combined and reassembled these codes under the sub-themes of participation of representatives of CSOs, and academic institutions and experts in COVID-19 preparedness and response in general and government-appointed taskforces and working groups in particular. During the third and final round of analysis, these sub-themes were reorganized under two broad rubrics of the extent and character of participation of representatives of CSOs and academic experts in COVID-19 governance.

#### Ethical approval

Approval to conduct the study was provided via Georgetown University’s Institutional Review Board (Ref: STUDY00005099; the study was determined to be exempt from full committee review).

## Results

### Extent of participation of academia and CSOs in COVID-19 governance

The extent of participation varied significantly across the six countries studied. While academia and CSOs were involved by the governments at least to some extent in all countries, they were mainly engaged to provide background support for decision-making or disseminating the decisions and policies. In rare cases, they were invited to the decision-making tables.

CSO participation in pandemic preparedness and response was reported to be mostly independent of the government’s actions in most countries, especially during the initial waves of the pandemic. In the subsequent waves, governments involved CSOs based on their presence in catering to vulnerable populations and their past relationships with the government. CSOs’ involvement also varied based on the existing approach and outlook of the government towards the civil society partners. For example, in Nigeria, while there was a strong willingness from CSOs and NGOs to contribute to the COVID-19 response, their involvement was not integrated with the government’s efforts. Some CSOs took the initiative independently to fill gaps in the government’s services, while others sought collaboration with the government but were met with unclear guidance or coordination mechanisms. ‘There were a lot of CSOs, a lot of NGOs, who wanted to do something. And some just went ahead and did what they felt was best. Others said [to the government], okay, we’re here, what do you want us to do’ (Nigeria 18).

Once the Nigerian government acted, NGOs that had existing formal collaborations with the government were first engaged. ‘So even before the COVID-19 outbreak, my NGO was part of the state mobilization committee. So, they [the government] just included me, and my NGO, in the risk communication, and community engagement pillar of the COVID response’ (Nigeria 17).

The same was true for involving the academic institutions ‘from the government side, we chose relevant institutions that were already doing research. We have, the Nigeria has the National Institutes for Medical Research… for medical research… from the academia (Nigeria 5).

Similarly, in Singapore, respondents alluded to the timely engagement of a few CSOs that have previously worked with the government, and typically with respect to engaging specific vulnerable groups.

‘Some of these groups have been in Singapore for the longest while dealing with advocacy and migrant rights… So with this, the various CSOs, those that were more trusted, were engaged more and were also used well [by the government]. So, they [CSOs] partnered to help the ministries and the government to push out messages, whether it is government helpline, or government health advisory’ (Singapore 17).

That said, the general lack of involvement of CSOs and NGOs in routine health governance and health systems functions in Singapore was overall mirrored by their limited participation in pandemic preparedness and response. ‘Civil society in Singapore …is relatively undeveloped… The government has not been particularly open to the appearance of lots of civil society organizations and NGOs’ (Singapore 23).

‘Because of the exigency of the situation, the potential severity of the threat, and the rapidity at which the virus spread…. I think the government was very focused on rapid action and implementation…and took a strategic decision that we don’t [involve community at that stage]’ (Singapore 23)

On the other hand, in Bangladesh, the involvement of NGOs grew as the pandemic evolved. NGOs were reported to be inactive in the initial phases because of poor funding, but were later involved by the government in piloting public health interventions for pandemic response.

‘I think at the initial stages, everybody was puzzled. If you consider the first quarter or the first couple of months, NGOs and the private sector were not on board really, as they didn’t have enough funds… Many small NGOs have had to shut down because of the money crisis, …But gradually, NGOs came on board, they started to work, specifically in the [geographic] areas they were serving… And also, for piloting programmes, like, how can the community workers screen out the COVID cases and refer them for COVID test?’ (Bangladesh 4)

The situation was different regarding academic institutions in all countries. For example, in Bangladesh, respondents mentioned a clear involvement of academic experts in the COVID-19 taskforces, ‘At the central level, it [involvement of academia] is structured, because the academicians, they [academic experts] were also part of the national advisory committee. They were [present] in the high-level meeting of [COVID-19] committees and sub-committees’ (Bangladesh 9)

Whereas, in Singapore, the presence of government research institutes blurred the line between government actors and academia. The collaboration between government and academic institutes stems from a longstanding partnership between the MOH and select academic institutions. The respondents reported a notable involvement of select academic institutions and experts who were invited to participate in a ‘working group committee that involved academia, the MOH in both research institutions in Singapore, hospitals and the National Center of Emergency’ (Singapore 11). ‘We had government research institutes coming to help us with analytics.’ (Singapore 5).

For the UK, the extent of community engagement and civil society involvement varied by region. ‘There’s no doubt that, in Wales and Scotland, there was far more community engagement, civil society involvement with the COVID response than in England’ (UK 20). There was also a concerted effort by the UK government to engage academic experts in addressing various issues related to the crisis. This engagement was facilitated through open calls made to academic experts through social media and other digital communication channels. However, the degree of involvement of academic experts in decision-making discussions is unknown.

‘I think it [government’s call to invite academics] went to all universities in the UK. It was a message from the Government office, and maybe, through social media, I think through LinkedIn… saying, we’re looking for all UK experts on issues related to COVID. Please fill in this Google form. It was very *ad hoc*.’ (UK 29)

However, it is not clear how the responses were managed or used, and therefore does not necessarily demonstrate active inclusion of academia by the government.

### Characterization of participation of academia and CSOs in COVID-19 governance

Academia’s participation in COVID-19 governance was often characterized by direct and formal involvement in government-appointed working groups and task forces focusing on specific policy issues, as well as indirect contributions by independently generating policy evidence through publications and other academic and non-academic outputs like media briefings. Academics from fields such as infectious diseases and biostatistics were particularly sought after. On the other hand, CSOs’ participation was largely limited to risk communication and service delivery, particularly in vaccine delivery.

The timing of participation also varied, with CSOs often being involved after reports of the spread of infection or low adherence to public policies. Academic experts’ engagement varied, ranging from engagement during the early preparedness phase to when there was an increase in caseload, followed by continued engagement throughout the response period, providing crucial insights and evidence-based recommendations.

#### Inclusion in governance structures and provision of expertise

These aspects of participation were more frequently observed with respect to academic partners across the countries we studied. We observed a frequent overlap between public research institutions and government entities in multiple countries, blurring the boundaries between (independent) academia and government. For example, in Nigeria, the government involved research institutes to generate evidence to support decision-making. These institutions were mostly medical research institutions from the public sector and a few independent academic experts.

‘…From the government side, we chose relevant institutions that were already doing research, or they had [earlier done research]. We have the Nigeria National Institute for Medical Research that does medical research. So, we included that. And then, the National Veterinary Research Institute was brought in. We brought in the environment [researchers] from the academia, as well. And then we looked at tertiary academic research institutes’ (Nigeria 5).

Discussing the composition of COVID-19 working groups and taskforces, interested parties in Nigeria expressed concern regarding the overemphasis and excessive presence of scientists and doctors that may not fully address the social and economic complexities of disease outbreaks.

‘If I look at the composition of the task forces… it is skewed towards scientists and doctors because we think these are people that were needed. It’s not right, because we know that infectious disease outbreaks, in fact, go beyond the health sector’ (Nigeria 6).

Singapore reported to have extensively involved academic experts throughout the pandemic, including in the preparedness phase. Academia was involved in reviewing data and providing evidence reports to inform policymaking.

‘So, there will be people in the Ministry of Health, including us, we will review the data, give inputs into policies, there also will be the academics who will give the inputs. And to make it more complex, the MOH engaged some of these academics to provide reports as well.’ (Singapore 13).

Specifically, the Singaporean government worked very closely with the author’s institute and Nanyang Technological University and involved other academic and research institutions as the outbreak evolved. With reference to the ‘working group committee’ that was formed across sectors: ‘[we] collectively came together to create the evidence and consults within the medical literature, and present [to the government] what was relevant to us’ (Singapore 11)

‘as we grappled with lack of information, the environment was new, new virus…we split responsibilities… we formed a working group committee that involve academia, involve the Ministry of Health, both research institutions in Singapore, and collectively came together to create the evidence’ (Singapore 18).

In the UK, in response to inadequate transparency in governments’ decision-making, senior academics and independent groups collaborated to create parallel initiatives to the government, like the establishment of an Independent Scientific Advisory Group for Emergencies (SAGE).

‘The UK is a small country, we all know each other, as academics, senior academics, whether you’re on SAGE or not… but in fact, when the minutes were kept secret… and it was clear that there were political members of the committee, not civil servants… and given as well that the government were really making the wrong decisions… we decided that we would establish an independent SAGE’ (UK 20)

Generally, across the countries studied, the participation of CSOs in COVID-19 policy-making and participation in governance bodies remained weak in comparison to those of academic experts and institutions, as exemplified by this respondent’s observations of Singapore:

‘Currently, the government’s running their own research to support the policy. And they’re partnering, for example with, established institutions such as universities and hospitals, but I think engagement with CSOs can be strengthened at this point’ (Singapore 16).

#### Technical support and service delivery

We observed a number of examples where academic institutions and CSOs collaborated closely with national governments on specific technical aspects of the response, or to deliver services. In some cases, the technical services were linked to inclusion in provision of expertise or participation in governance bodies, as described above, but in other cases, it was more focused on addressing a particular response need. For example, the Singaporean government partnered with a university to develop a digital mobile phone application, *TraceTogether*, for surveillance, verifying vaccination status and communication with people to provide them with information regarding case clusters.

‘I think that the ‘TraceTogether’ is the major kind of collaboration between academia and government, so you give safe entry to people [in public places]. So, we checked the vaccination status and tried to adjust the group size for gathering, etc.’ (Singapore 16).

South Africa adopted a proactive approach to leveraging existing academic and research expertise. However, this was dominated particularly by government-sponsored institutions with specific technical expertise, notably virology and genomic sequencing, involving research laboratories across the country providing technical services to monitor the spread of the virus.

‘Because we’re a small country, we know each centre’s expertise… for example, we knew that there’s a genomic sequencing [expertise] based in the University of KwaZulu Natal. And so, they were therefore involved… we were able to then set the National Genomic surveillance group quite early… and were able to ensure that we were actually sequencing samples across the country’ (South Africa 16)

Similarly, in Jordan, the Jordan University of Science and Technology (JUST) aided the government in COVID genome sequencing, ‘…The Princess Haya Biotechnology Centre at JUST decoded the genetic code for the emerging coronavirus through next-generation sequencing technology. We [the scientists at JUST] were among the first to, I think, isolate and detect the British strain [sic] in Jordan’ (Jordan 3)

CSO participation in response efforts was conducted both independently and in support of government actions; for the latter, governments involved CSOs in a range of activities, including risk communication, awareness generation, conducting polls to understand people’s response to the policies, as well as distribution of services and goods like face masks, sanitizers, drugs, and vaccines. In Bangladesh, various CSOs and NGOs played a crucial role in the implementation of COVID-19 programmes.

‘They [CSOs] provided the sanitizers and did awareness activities; NGOs also worked in the city corporation area during our COVID-19 vaccination programme. They provided their manpower, specifically for vaccination programmes’ (Bangladesh 4).

In Singapore, selected NGOs were involved by the government for risk communication and spreading information about government policies to migrant populations.

‘…the various CSOs, those that were more trusted, were engaged more, and were also used well. So, they partnered to help the ministries and government push out messages, whether it is government helpline, or government health advisory and things like that. (Singapore 18).

## Discussion

The COVID-19 pandemic highlighted the necessity for inclusive governance, especially the participation of CSOs and academic experts in decision-making during health emergencies. Our comparative research across six nations—Nigeria, Singapore, South Africa, Bangladesh, Jordan, and the UK—examined the interactions, deficiencies, and possible pathways for integrating these perspectives into governance and decision-making processes during health emergencies. We observed that the extent and nature of participation of CSOs and academia varied across all six selected countries, and built on existing governance models and the history of involving diverse stakeholders in the governance mechanism. For example, Nigeria’s use of organized engagement with selected civil society and academic institutions in limited task forces stems from its existing relationship with these organizations built over the previous disease outbreaks as well as for managing other health issues (Ngara 2019, Asiyanbi and Towoju 2025, Ibrahim and Ismail 2016). Likewise, Singapore’s reliance on established government research collaborations and late involvement of NGOs to support the outbreak among migrant population results from its chronic neglect of CSOs and a centralized governance model (Asthana et al. 2024; Kim et al. 2022), whereas South Africa’s efforts to bring CSOs into service delivery are a replication of its governance model used in the HIV/AIDS epidemic. Its use of genomic experts in pandemic response builds on efforts to expand genomics capacity for combating drug-resistant HIV-1 and tuberculosis through precision medicine and genomic epidemiology (GenPath Africa 2025; Coutinho et al. 2012).

These findings complement the research from China, Japan, South Korea and Taiwan, showcasing CSOs collaboration with governments in quarantine measures, logistical support, advocacy for marginalized groups, organizing community responses, distributing essential supplies, and conducting screening programmes (Huang 2020; Cai et al., 2021; Choi 2020). In low- and middle-income countries, CSOs have been crucial in addressing the needs of racialized minorities, including refugees, by supplementing government emergency aid responses with essential resources and support for highly vulnerable populations (Nourpanah 2021). These efforts illustrate the potential of CSOs to act as intermediaries between governments and communities, particularly in settings where state capacity is limited or services are provided discriminatorily (Golechha 2020; Ahmed et al. 2023; Levine et al. 2023; Mohseni et al., 2022).

Our research confirms other studies that highlight the predominance of biomedical experts (Colman et al. 2021), virologists and epidemiologists and non-governmental participants in the COVID-19 response, noting that while CSOs played a significant role during the pandemic, they were often excluded from decision-making bodies (Rajan et al. 2020), depriving them of the opportunity to contribute expertise and experience in managing a public health crisis. The issue of CSOs’ exclusion in pandemic management is not new but has often been overlooked or misunderstood during past health crises, particularly the Ebola outbreak in West Africa. Evidence shows that international NGOs like Partners In Health and Wellbody Alliance (a local NGO) played significant roles in strengthening the health system and resuming the delivery of regular health services during an Ebola outbreak in Sierra Leone (Cancedda et al. 2016). Furthermore, the failure to engage community leaders resulted in long-term challenges, including widespread mistrust and violence during this outbreak (Arthur et al. 2022). Despite these lessons, NGOs and other local leaders were not adequately included for information sharing and provision of guidance to support COVID-19 pandemic management.

We found that the understanding of academia’s role in public health decision-making has not been adequately studied, especially in comparison to CSOs, development partners, and the private sector. Our findings align with results from Colombia, showing the role of academic initiatives in translating scientific knowledge into pandemic policies (Turner et al. 2021). Furthermore, we confirm the findings from other European countries indicating the dominance of biomedical sciences and under representation of perspectives from economics, sociology, and anthropology in COVID-19 policy-making (Hodges et al. 2022). This exclusion of interdisciplinary expertise is a missed opportunity, as evidence-based decision-making relies on diverse insights. For example, anthropologists, sociologists, and economists can significantly contribute to governance by leveraging their expertise in community behaviours and analyzing the social and financial impacts of pandemic policies (Gupta and Sengupta 2021).

We make four broad recommendations to improve the time, extent, and process of participation of CSOs and academia in future pandemic governance. First, governments should incorporate diverse expertise from CSOs and academia into the governance frameworks. This includes integrating CSOs working at national, sub-national, and local levels, particularly those working with marginalized populations and having a presence in hard-to-reach areas. Similarly, expertise from social sciences should be integrated to enrich the decision-making processes. Second, the governments should emphasize the early involvement of CSOs and academic experts in crisis situations. Engaging these groups from the preparedness phase of disease outbreaks can effectively leverage their expertise in crafting proactive policies that address potential health threats before they escalate. Third, governments should institutionalize the participation of CSOs and academia by establishing formal mechanisms such as reserving seats for experts from diverse disciplines in emergency task forces and working groups. These mechanisms should operate in both peace time and crisis situations to ensure continuous input and preparedness. Lastly, multi-sectoral collaboration should be strengthened as part of routine health governance. Building robust partnerships between government entities and non-governmental actors, such as academia and CSOs, for routine decision-making will ensure that the partnerships can be effectively utilized in health emergencies.

Our research was not without its limitations. One significant challenge we encountered in designing this study was distinguishing the roles of interest holders in the overall management, governance, and decision-making related to COVID-19. Though we examined participation through government-appointed decision-making taskforces and working groups, we recognise that this does not fully address the issue of inclusion but rather reflects their representation only, which, while necessary, is insufficient to ensure full participation in governance. Our other challenge related to the timing of various pandemic waves in the selected countries in our analysis. We collected data in phases across these countries, which restricted our ability to analyse the participation of CSOs and academia longitudinally or comparatively during different periods within each country or between them. Furthermore, while we studied the role of multiple parties in COVID-19 decision-making, for this analysis we focused on the roles of CSOs and academia due to their growing influence in decision-making and pandemic governance. This choice meant we could not explore in detail the contributions of other significant interest groups, such as the private sector and development partners, which could have offered a more comprehensive understanding of the governance process. We also recognise the limitations of qualitative interview-based methods of data collection, as they reflect the perspectives of a select few study participants, but we value the depth of data gained from their sharing of life experiences.

In conclusion, this study contributes to the practice of pandemic governance by presenting the extent, nature, and timing of the participation of CSOs and academia in governance and decision-making processes across six countries. The findings underscore the need for institutionalizing collaboration, incorporating diverse expertise, and ensuring early and meaningful involvement of interested parties. Future research should focus on participatory and inclusive decision-making processes to address the concerns of tokenism and biases in participant selection and engagement.

## Data Availability

The data underlying this article will be shared on reasonable request to the corresponding author.

## Reflexivity statement

This study was grounded in equal partnership and strong involvement from low- and middle-income country (LMIC)-based researchers throughout the study. Local team members (W.U., A.C.I., I.B.G., J.J.W., M.M.H., T.S., C.W., N.G., and M.A.N.) played key roles in shaping the study design, identifying participants, collecting data, and validating country-level findings. The study addressed pressing local priorities related to governance and decision-making deficits in pandemic preparedness and response. Funding from the US CDC supported contributions from Nigeria-based researchers (W.U., A.C.I., I.B.G.). Early-career researchers (R.L., R.V.M.) received mentorship and training in qualitative methods, and LMIC researchers in Nigeria gained experience in governance and emergency decision-making research methods. Authorship reflects contributions across all career stages, with student researchers and LMIC team members acknowledged as co-authors. While the authorship team includes more members from high-income countries—due to the location of postdocs and students involved—LMIC researchers led key components of the work. The team also maintained gender balance, with ten authors identifying themselves as women and six identifying as men. Although the project did not directly enhance physical infrastructure, it contributed to local capacity and is expected to inform policy and future pandemic preparedness efforts through targeted dissemination.
